# Supplementary material for: Orientation distributions of vacuum-deposited organic emitters revealed by single-molecule microscopy
Source: Nat Commun. 2023 Oct 2;14:6126. doi: 10.1038/s41467-023-41841-2 (PMC10543598; doi:10.1038/s41467-023-41841-2)
Supplement: Supplementary file 1 — Supplementary Information [file 41467_2023_41841_MOESM1_ESM.pdf]

Supplementary Information

Orientation distributions of vacuum-deposited organic emitters revealed by single-molecule microscopy

Francisco Tenopala-Carmona[1,2,], Dirk Hertel[1], Sabina Hillebrandt[1], Andreas Mischok[1], Arko Graf[2], Philipp Weitkamp[1], Klaus Meerholz[1], Malte C. Gather[1,2,*]*

1. Humboldt Centre for Nano- and Biophotonics and Institute of Physical Chemistry, Department of Chemistry, University of Cologne, Greinstr. 4-6, 50939 Köln, Germany.
2. Organic Semiconductor Centre, SUPA School of Physics and Astronomy, University of St Andrews, St Andrews, KY16 9SS, UK.

*Email: Malte.Gather@uni-koeln.de, F.TenopalaCarmona@uni-koeln.de

Supplementary Methods

An ensemble of dipoles that follows an isotropic distribution can be represented by a uniform distribution of points over the unit sphere. This distribution of points scales proportionally to the surface area as a function of the polar angle theta, i.e., $P(\theta) \propto \sin(\theta)$. This can be seen in **Supplementary Figure 1a**, which shows an array of 2000 random points uniformly distributed over the unit sphere. By contrast, **Supplementary Figure 1b** shows an array of 2000 points normally distributed over the same sphere, with a mean value of 31.23° and a standard deviation of 11.46°. The histograms shown in **Figure 1a** of the main text follow the same distributions, but contain data from 100,000 points. All hypothetical distributions were generated using the software Matlab using the functions *rand* for uniformly distributed points or *normrnd* for normally distributed points).

The experimental orientation distributions were obtained from the single-molecule defocused orientation and position imaging (DOPI) data as follows. First, we used optical models from the Matlab package QDControl,[1] which follows the theory described by Böhmer and Enderlein in Ref. [2], to generate a library of *r* defocused patterns:

$$P^{(r)} = P^{(r)}(x, y, z, \theta, \phi, dz, d, n_{host}, n_{substrate}, NA, M, \lambda) \qquad (1),$$

where *x*, *y*, and *z* are the Cartesian coordinates of the transition dipole within the film, θ and φ are the polar and azimuthal orientation angles, *dz* is the out-of-focus distance, *d* is the thickness of the film, n_{host} and $n_{\mathrm{substrate}}$ are the refractive indices of the film and the substrate, respectively, *NA* is the numerical aperture of the microscope objective (1.45), *M* is the total magnification of the system (280×), and λ is the wavelength of the emitted light (520 nm due to the narrow band-pass filter in the imaging path). *d*, n_{host} and $n_{\mathrm{substrate}}$ were obtained from ellipsometry measurements and *dz* was controlled using the Perfect Focus System of the microscope, which allows accurate control of the position of the objective with respect to the substrate. For the experiments done in PMMA, z, θ, ϕ were set to vary in the pattern library. For thermally evaporated samples, *z* was fixed at the known position of the molecules and θ, ϕ were varied. In order to fit the *z*-position of each molecule, we first needed to determine the in-focus position of the sample (i.e., *dz* = 0). This was achieved by comparing the patterns from the brightest molecules to optical simulations using a modified version of the software QDControl. The

selected value was then fixed for all other identified patterns in the same dataset (525 nm for the solution-processed samples, and 500 nm for the experiments for the thermally-evaporated samples).

Next, we followed the algorithm introduced by Patra and Enderlein[3] to calculate the minimum error for each pattern from the library centred at each pixel (m,n) of the experimental image:

$$e_{mn}^{(r)} = \sum_{j,k} s_{jk} \left(x_{m+j,n+k} - c_{mn}^{(r)} p_{jk}^{(r)} - d_{mn}^{(r)} b_{jk} \right)^2 \qquad (2),$$

where j and k denote the pixels of a subsection of the image centred at pixel m,n, s is a circular mask consisting of values 1 and 0 only, c is a constant that accounts for the brightness of each pattern, b is a uniform normalised background value¸ and d is a scaling factor for the background. From this calculation, the minimum value of $e_{mn}^{(r)}$ at each pixel m,n, $\tilde{e}_{mn}$, is identified, and thereby the best-fitted pattern $P^{(r)}$ for that pixel is selected. Then, these values are used to generate a map of the inverse minimum error for each pixel weighted by the brightness of the corresponding pattern, $L_{mn} = \tilde{c}_{mn}/\sqrt{\tilde{e}_{mn}}$. Thresholding of this map leads to the identification of the position of the molecules in the image.

However, as pointed out by Patra and Enderlein, due to the symmetry of the defocused patterns for wide polar angles (see **Figure 1c** of the main text), single defocused patterns lying close to the horizontal can be misidentified as two independent patterns with values of θ close to the vertical. Therefore, we implemented an additional segmentation algorithm available at [4], which is described below and illustrated in **Figure S2**.

1. Thresholding of the inverse error map L_{mn} with low threshold values (0.5).
2. Calculation of a distance-transformed map of the pixels above threshold.
3. Filtering of the distance-transformed map using a 2D median filter.
4. Segmentation using a Watershed transform.

From that segmentation, each cluster of pixels above threshold was treated as a region belonging to the same identified molecule. Then, the maximum value of L_{mn} was identified, its corresponding pixel was selected as the best fitted position for that molecule, and the corresponding values of $P^{(r)}$, $\tilde{c}_{mn}$, and $d_{mn}^{(r)}$ were selected as its best fitted pattern, brightness, and background, respectively. Finally, a threshold value for L_{mn} depending on the signal-to-noise ratio of the specific measurement was selected and patterns below that threshold were discarded. This threshold was set to 1.0, 0.7, and 1.8 for the experiments in PMMA, on bare glass, and in mCP, respectively.

The error in each of the free-fitting parameters ($\xi = \theta, \varphi, z$) was estimated from the inverse-square error of each fit as

$$\Delta\xi = \xi^{(r)} - \xi^{(r+l)} + \Delta\xi_{min} \qquad (3),$$

where $\Delta\xi_{min}$ is the error related to having a minimum step size for each parameter in the pattern library, and l indicates the lth-nearest neighbour to the best-fitted pattern $p^{(r)}$ in the library such that

$$e_{mn}^{(r)} = \sum_{j,k} s_{jk} \left(x_{m+j,n+k} - c_{mn}^{(r)} p_{jk}^{(r)} - d_{mn}^{(r)} b_{jk} \right)^2 < c_{mn}^{(r)} \sum_{j,k} s_{jk} \left(p_{jk}^{(r)} - p_{jk}^{(r+l)} \right)^2 \qquad (4).$$

In this way, the estimated error in each parameter ξ satisfies the condition that the squared difference between the patterns $p^{(r)}$ and $p^{(r+l)}$ is larger than the squared difference between the best-fitted pattern and the experimental data. $\Delta\xi_{min}$ was set to the minimum step size of each parameter's grid unless $\xi^{(r+l)}$ coincided with the boundaries set for each parameter (0° and 90° for θ, and 0 nm and 30 nm for z in the experiments involving solution-processed samples), in which case $\Delta\xi_{min}$ was set to ½ of the minimum grid step. The error in the values of a obtained from single-molecule orientation distributions was calculated directly from error propagation using the estimated error in θ for each identified molecule.

Supplementary Figures

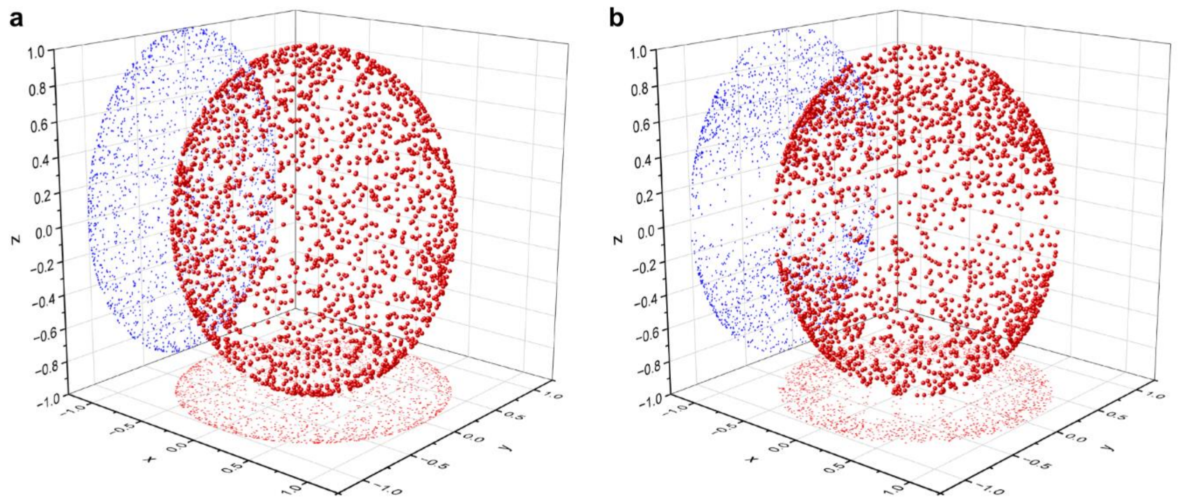

Supplementary Figure 1. Graphical representation of orientation distributions. a, isotropic and **b**, non-isotropic distributions. Both have the same anisotropy factor $a = \langle \cos^2\theta \rangle = 0.33$. Source data are provided as a Source Data file.

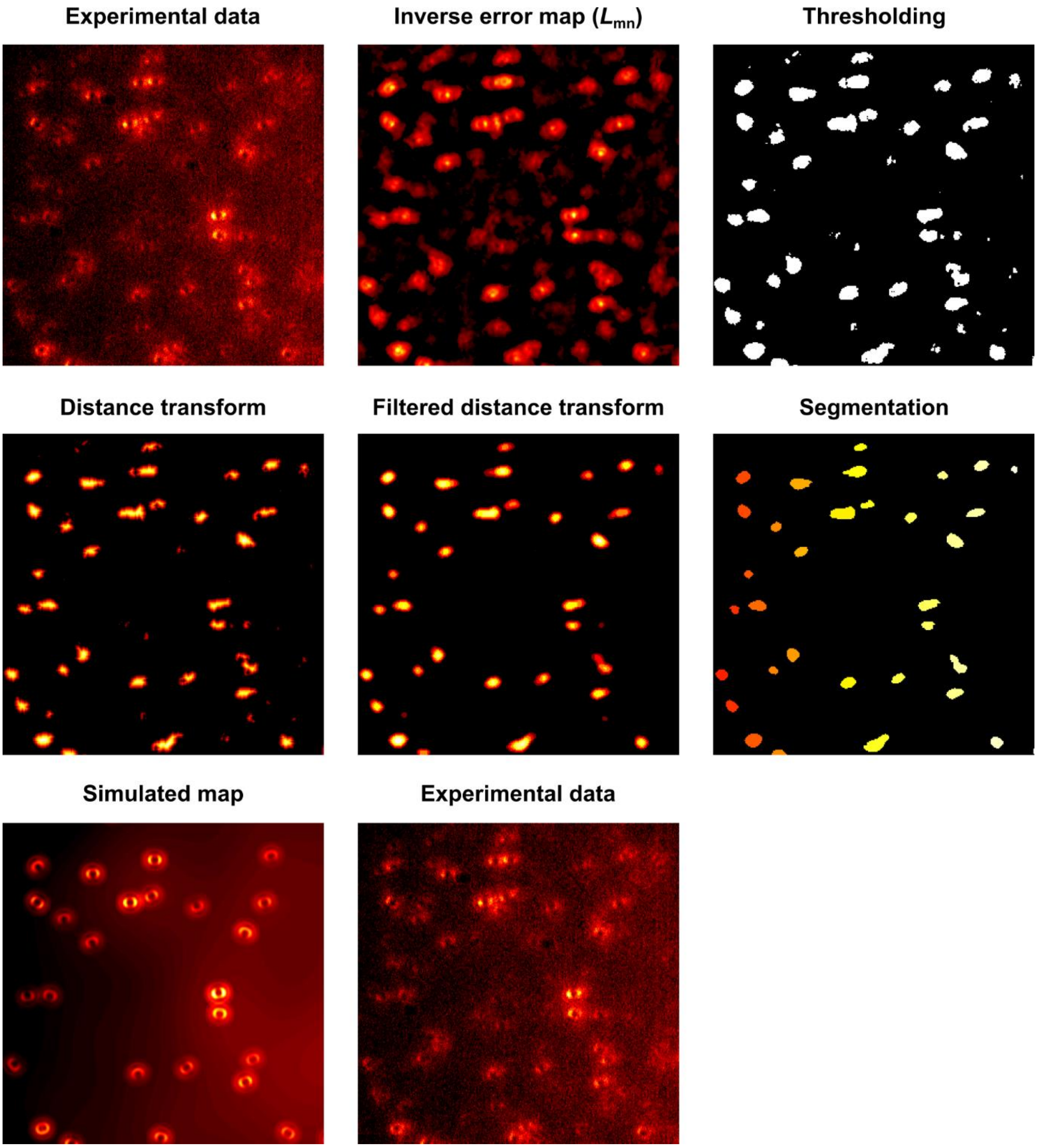

Supplementary Figure 2. Graphical description of the segmentation algorithm used for DOPI data analysis. The experimental data is shown twice for direct comparison with the simulated and inverse-error maps. For visualisation purposes only, a uniform background was subtracted from the experimental data to improve contrast.

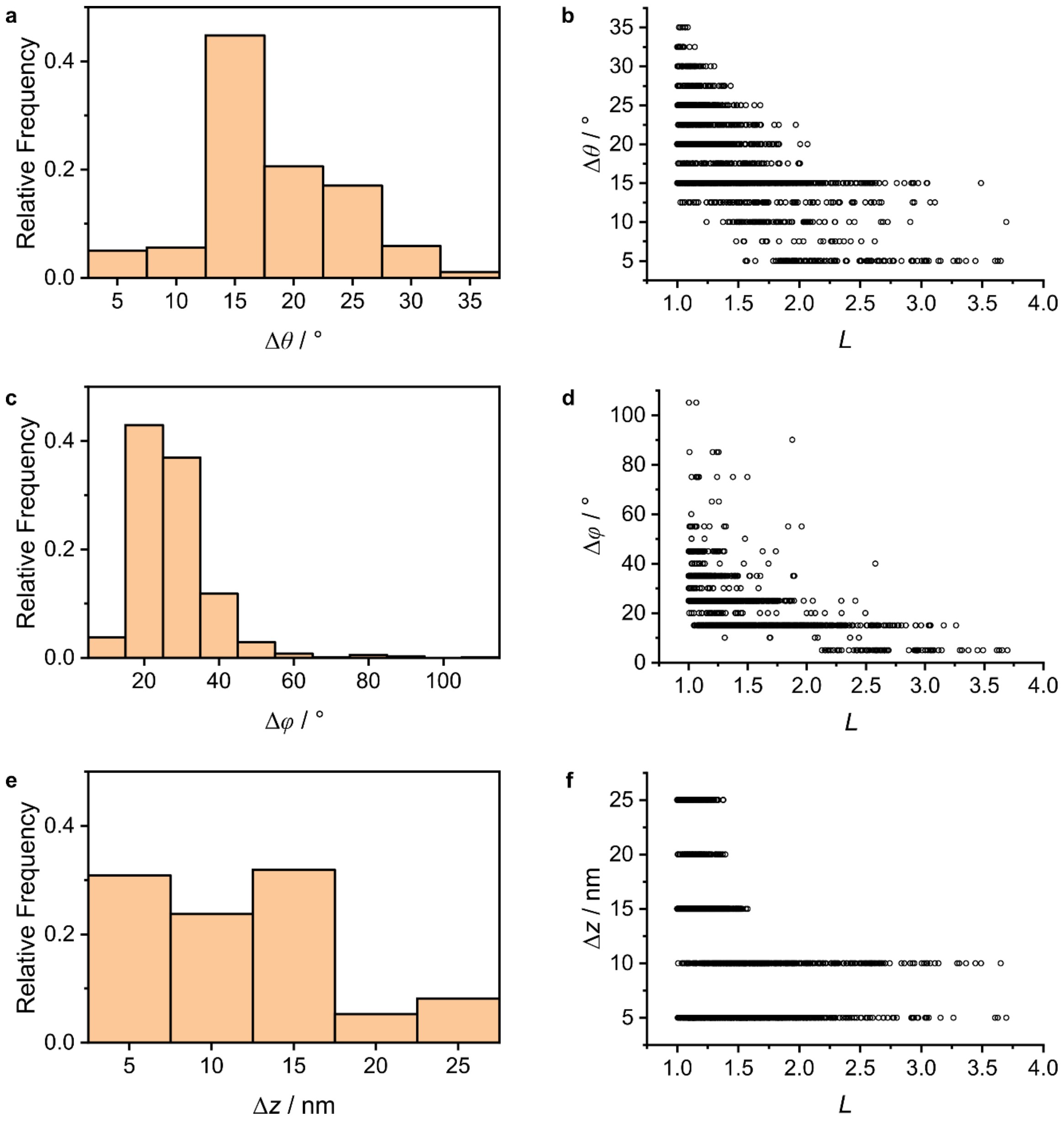

Supplementary Figure 3. Histograms of the distributions of errors and scatter plots of the error values against the weighted inverse minimum error (*L*) for the free-fitting parameters *θ* (a, b), *φ* (c, d), and *z* (e, f) of C545T molecules dispersed in PMMA. The error in the determination of these parameters decreases with increasing values of *L*. Only the patterns satisfying the threshold condition for this sample $L \geq 1.0$ were included in these plots.

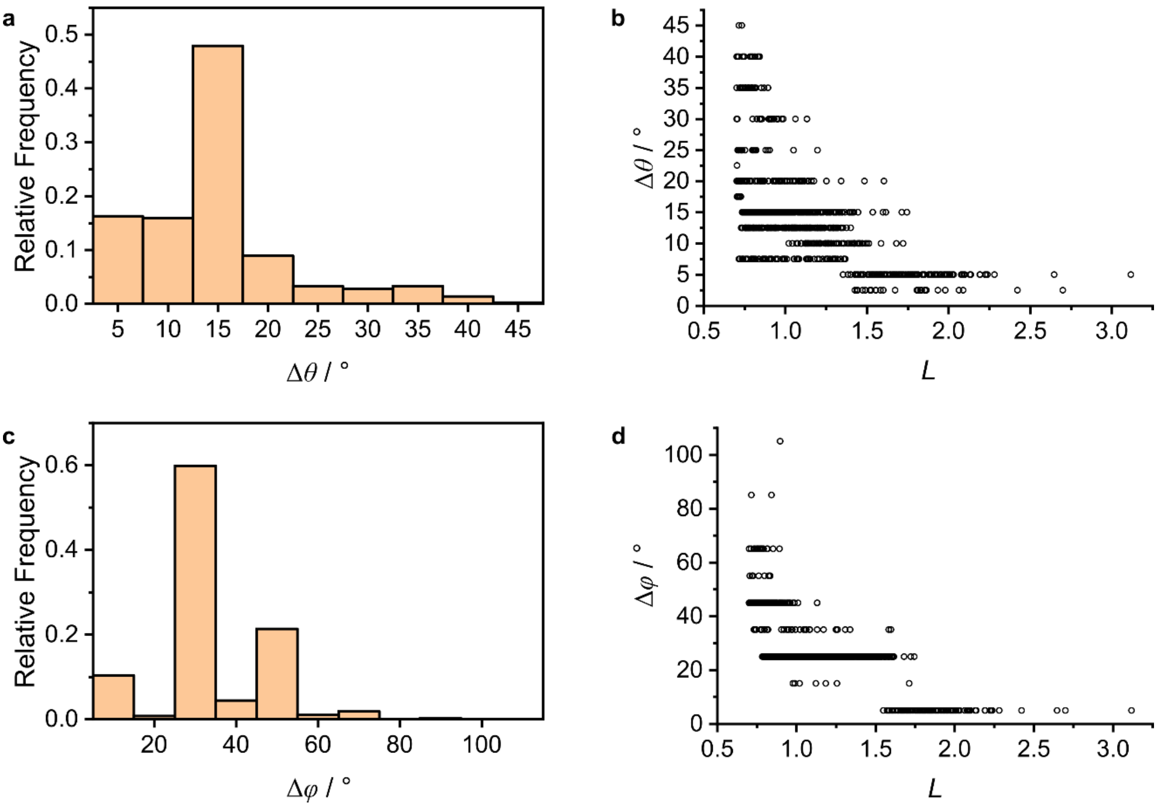

Supplementary Figure 4. Histograms of the distributions of errors and scatter plots of the error values against the weighted inverse minimum error (*L*) for the free-fitting parameters θ (a, b) and φ (c, d) of C545T molecules deposited on bare glass. Only the patterns satisfying the threshold condition for this sample $L \geq 0.7$ were included in these plots.

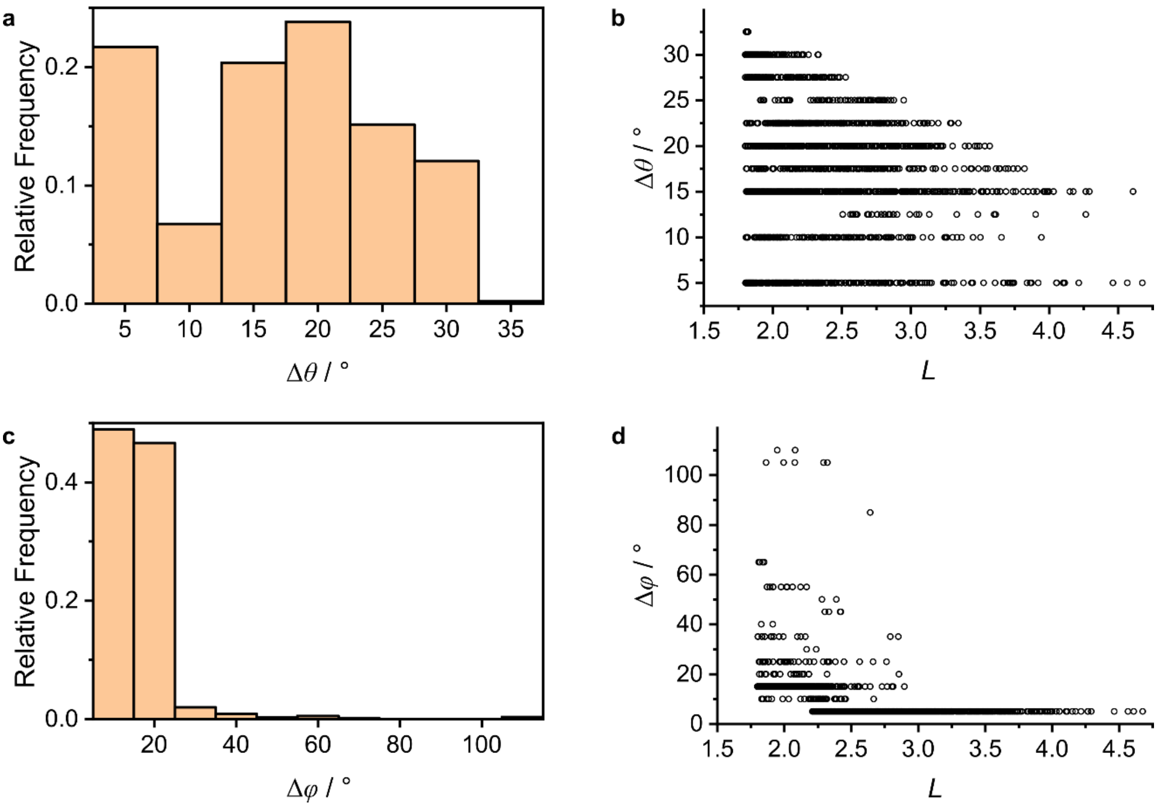

Supplementary Figure 5. Histograms of the distributions of errors and scatter plots of the error values against the weighted inverse minimum error (*L*) for the free-fitting parameters *θ* (a, b) and *φ* (c, d) of C545T molecules dispersed in thermally evaporated mCP. Only the patterns satisfying the threshold condition for this sample $L \geq 1.8$ were included in these plots.

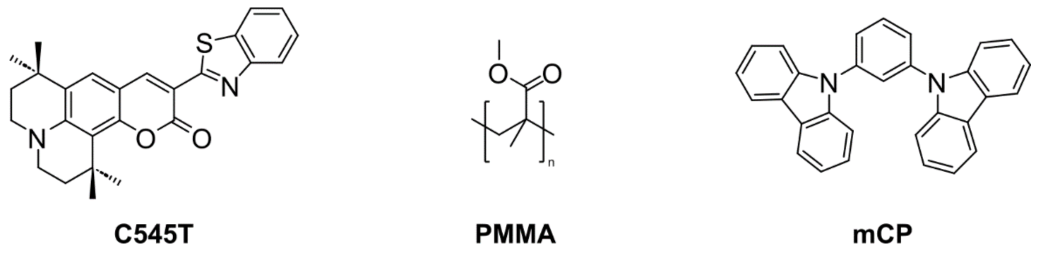

Supplementary Figure 6. Chemical structures of the materials used in this work.

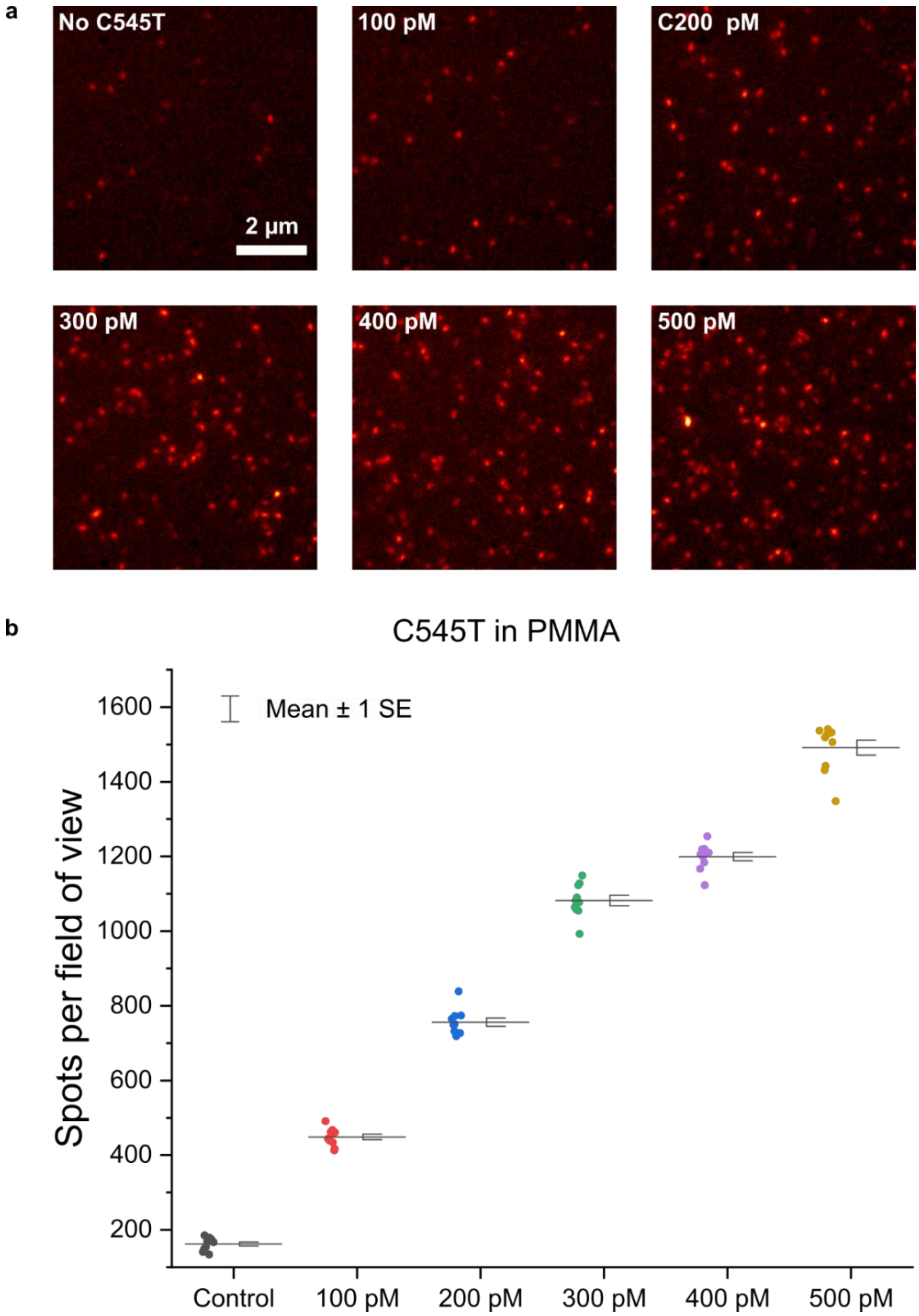

Supplementary Figure 7. **Measurement of number of molecules per field of view in samples for single-molecule microscopy. a**, Fluorescence microscopy images of spin-coated samples of C545T in PMMA. The label indicates the concentration of C545T in the coated dilution. For visualisation purposes only, a uniform background was subtracted to improve contrast. **b**, Number of molecules per field of view found in the corresponding solution-processed samples. The control sample is made of PMMA only (no C545T).

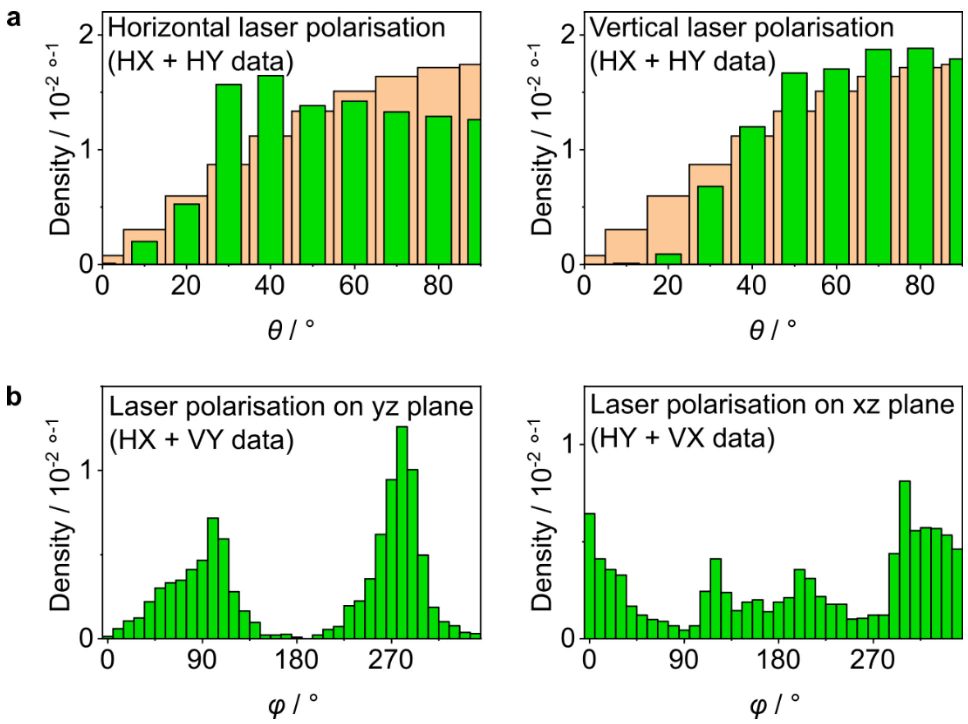

Supplementary Figure 8. **Correlation of the measured TDM orientation angles and the different polarised excitation conditions**. **a**, Distribution of polar angles for the combined datasets measured using HX + HY (horizontal) excitation and VX + VY (vertical) excitation. The distribution is shifted towards smaller or larger angles for vertical and horizontal excitation, respectively. **b**, Distribution of azimuthal angles for the combined datasets measured using HX + VY (excitation parallel to the *yz* plane) and HY + VX (excitation parallel to the *xz* plane), respectively. The peaks in the distributions are located at complementary positions.

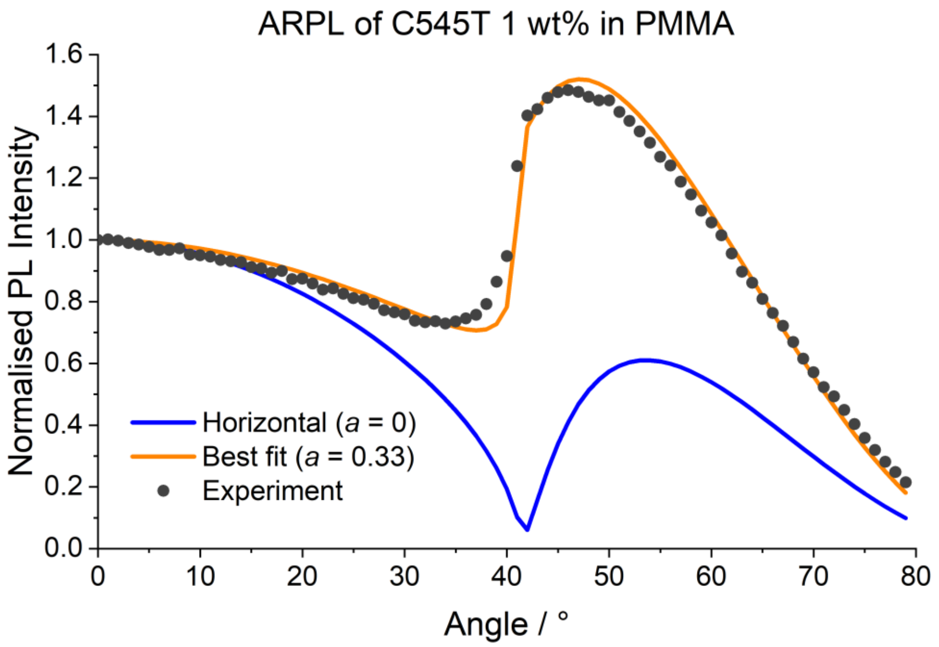

Supplementary Figure 9. Angle-resolved photoluminescence measurements of spin-coated C545T dispersed at 1 wt% in PMMA. Source data are provided as a Source Data file.

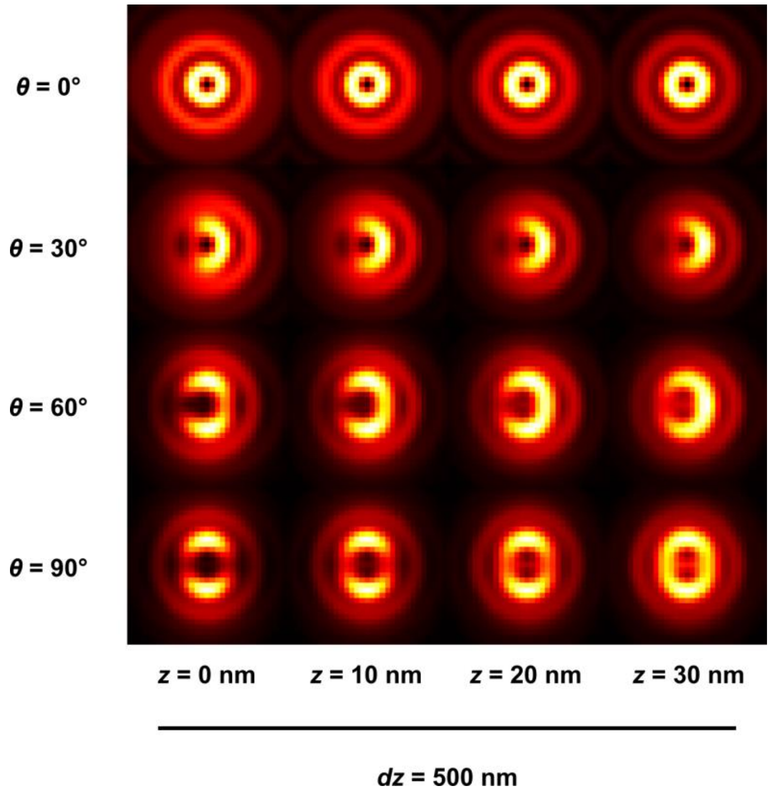

Supplementary Figure 10. Optical simulations of defocused orientation patterns as a function of the polar angle TDM of the molecule and its *z*-position within the film. Defocussing *dz* was 500 nm for all simulations.

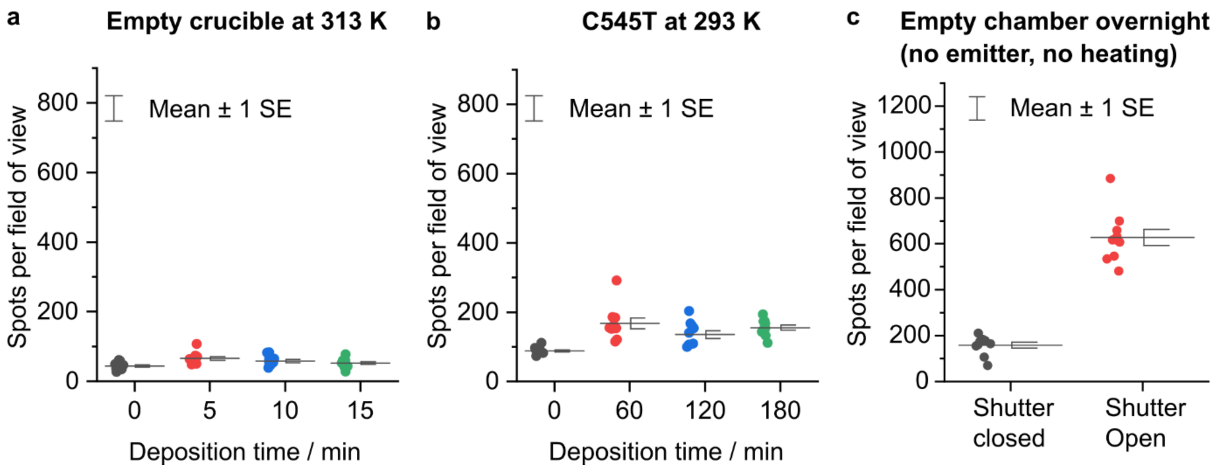

Supplementary Figure 11. **Control tests for thermally evaporated samples of C545T on bare glass. a**, Quantification of the base level of fluorescent impurities by mapping the number of emissive spots per field of view (~40×40 μm^2) in a control run with substrates exposed to **a**, a heated but empty crucible, **b**, a crucible with C545T with no active heating, and **c**, the evaporation chamber in high vacuum overnight with no crucibles nor active heating. There is no correlation between the number of spots and the exposure time for periods within the duration of our emitter deposition processes for the samples used in single-molecule microscopy measurements. Furthermore, the impurity level in this range is well below the target concentration in **Figure 3b** of the main text, and it only becomes more significant for long exposure times (panel **c**).

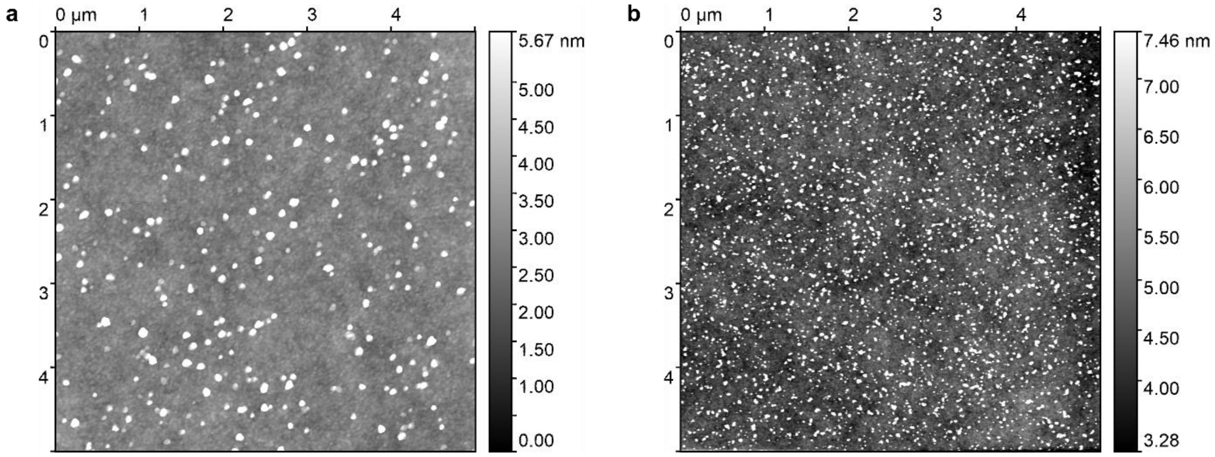

Supplementary Figure 12. Atomic force microscopy measurements of as-cleaned substrates. **a**, substrates used for thermally evaporated samples (RMS roughness: 2.14 nm). **b**, substrates used for solution-processed samples (RMS roughness: 1.33 nm). These RMS roughness values are in the order of the length of the C545T molecule which is 1.55 nm.[5]

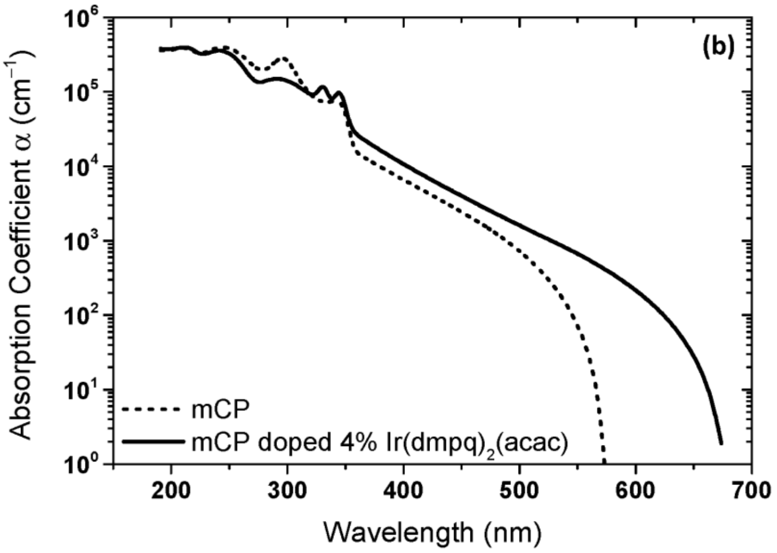

Supplementary Figure 13. Absorption spectrum of mCP with absorption coefficient shown on a logarithmic scale. Reproduced from Tselekidou, D., Panagiotidis, L., Papadopoulos, K., Kyriazopoulos, V. & Gioti, M. A Comparative Study of Ir(dmpq)$_2$(acac) Doped CBP, mCP, TAPC and TCTA for Phosphorescent OLEDs. *Photonics* **9**, 800 (2022) under the Creative Commons Attribution (CC BY) license (https://creativecommons.org/licenses/by/4.0/).

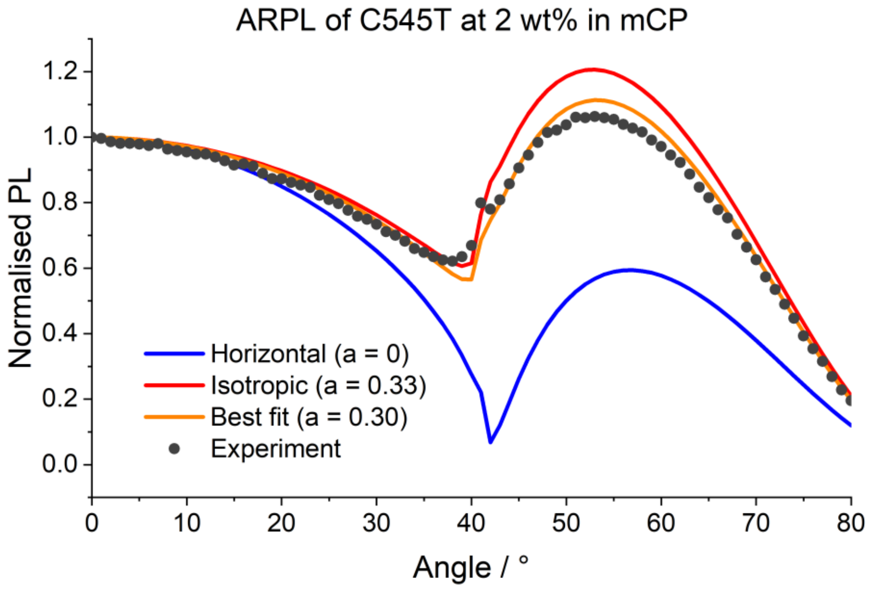

Supplementary Figure 14. Angle-resolved photoluminescence measurements of C545T dispersed at 2 wt% in mCP. Source data are provided as a Source Data file.

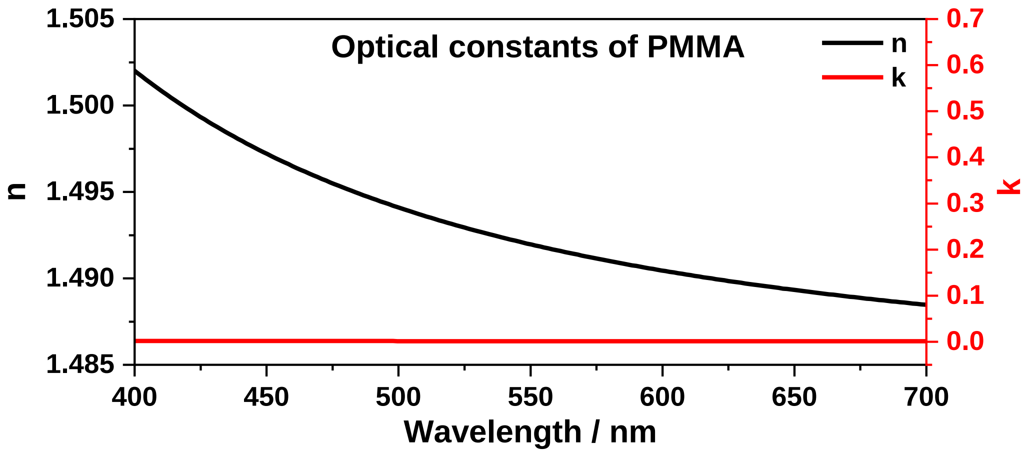

Supplementary Figure 15. Optical constants of PMMA. No significant improvement in fit quality over what is shown here was obtained when fitting the ellipsometry data to a birefringent model of the optical constants. Source data are provided as a Source Data file.

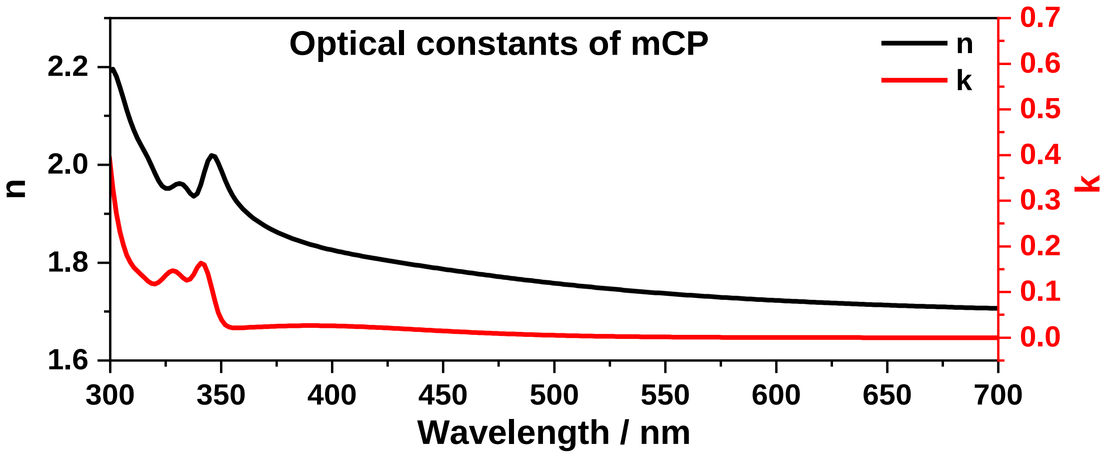

Supplementary Figure 16. Optical constants of mCP. No significant improvement in fit quality over what is shown here was obtained when fitting the ellipsometry data to a birefringent model of the optical constants. Source data are provided as a Source Data file.

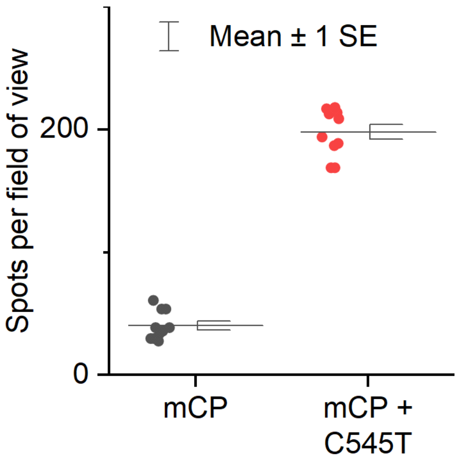

Supplementary Figure 17. Number of emissive molecules per field of view found in thermally evaporated samples for single-molecule microscopy. The control sample (mCP) was covered during the deposition of the emitter layer using adjustable shutters and consists only of a 40 nm layer of purified host material. The "mCP + C545T" sample was fabricated as depicted in **Fig. 4d** of the main text.

Supplementary References

1. Imaging of single molecules - Georg-August-Universität Göttingen. https://www.uni-goettingen.de/en/512319.html.

2. Böhmer, M. & Enderlein, J. Orientation imaging of single molecules by wide-field epifluorescence microscopy. *Journal of the Optical Society of America B* **20**, 554–559 (2003).

3. Patra, D., Gregor, I. & Enderlein, J. Image Analysis of Defocused Single-Molecule Images for Three-Dimensional Molecule Orientation Studies. *J Phys Chem A* **108**, 6836–6841 (2004).

4. Tenopala-Carmona, F. *et al.* Orientation distributions of vacuum-deposited organic emitters revealed by single-molecule microscopy (datset). *University of St Andrews Research Portal* (2023) doi:https://doi.org/10.17630/c835efe3-402d-4a9d-8ea0-afc5bad6efd2.

5. Tenopala-Carmona, F. *et al.* Identification of the Key Parameters for Horizontal Transition Dipole Orientation in Fluorescent and TADF Organic Light-Emitting Diodes (dataset). *University of St Andrews Research Portal* (2021) doi:https://doi.org/10.17630/ae55e842-6db8-4d3b-975c-b1905a0a45f3.
